# Support Vector Machine-Based Global Classification Model of the Toxicity of Organic Compounds to *Vibrio fischeri*

**DOI:** 10.3390/molecules28062703

**Published:** 2023-03-16

**Authors:** Feng Wu, Xinhua Zhang, Zhengjun Fang, Xinliang Yu

**Affiliations:** Hunan Provincial Key Laboratory of Environmental Catalysis & Waste Regeneration, College of Materials and Chemical Engineering, Hunan Institute of Engineering, Xiangtan 411104, China

**Keywords:** classification model, support vector machine, toxicity, *Vibrio fischeri*

## Abstract

*Vibrio fischeri* is widely used as the model species in toxicity and risk assessment. For the first time, a global classification model was proposed in this paper for a two-class problem (Class − 1 with log1/IBC_50_ ≤ 4.2 and Class + 1 with log1/IBC_50_ > 4.2, the unit of IBC_50_: mol/L) by utilizing a large data set of 601 toxicity log1/IBC_50_ of organic compounds to *Vibrio fischeri*. Dragon software was used to calculate 4885 molecular descriptors for each compound. Stepwise multiple linear regression (MLR) analysis was used to select the descriptor subset for the models. The ten molecular descriptors used in the classification model reflect the structural information on the Michael-type addition of nucleophiles, molecular branching, molecular size, polarizability, hydrophobic, and so on. Furthermore, these descriptors were interpreted from the point of view of toxicity mechanisms. The optimal support vector machine (SVM) model (*C* = 253.8 and *γ* = 0.009) was obtained with the genetic algorithm. The SVM classification model produced a prediction accuracy of 89.1% for the training set (451 log1/IBC_50_), of 80.0% for the test set (150 log1/IBC_50_), and of 86.9% for the total data set (601 log1/IBC_50_), which are higher than that (80.5%, 76%, and 79.4%, respectively) from the binary logistic regression (BLR) model. The global SVM classification model is successful, although it deals with a large data set in relation to the toxicity of organics to *Vibrio fischeri*.

## 1. Introduction

With the extensive application of chemicals such as pesticides, insecticides, chemical fertilizers, medicines, detergents, anti-foaming agents, and flocculants, their potent toxicity and organic natures have attracted more and more attention [1,2]. Among the 84,000 chemicals on the market, only 1% of them have been measured for safety. Furthermore, 1000–2000 new chemicals enter the market every year [3,4]. In addition to the criticism over animal testing, to test the toxicity and risk of chemicals is non-trivial and is often very time consuming [5].

Quantitative structure–activity/toxicity relationship (QSAR/QSTR) models have been proposed to assess the risk of chemicals [5]. QSPR approaches have the advantages that they need only the information of the molecular structure and do not rely on any empirical data. The QSPR principle is based on the assumption that it is the molecular structure that is determining the physical and chemical properties of molecules, and that compounds possessing similar molecular structures have similar properties. The molecular structure (i.e., the three-dimensional structure or arrangement of atoms in a molecule) is governed by several factors, including covalent bonds and polarity, non-covalent interactions, the hydrophobic effect, shape and size, and so on. It can be quantitatively described by molecular descriptors related to the physical and chemical properties of molecules.

*Vibrio fischeri* is a bioluminescent and widely used as the model species since it is sensitive to a wide range of toxicants [5]. There are several QSTR models reported for the assessment of the aquatic toxicity of chemicals against *Vibrio fischeri*. For example, Cronin and Schultz [6] developed three single-descriptor (logKow) models of toxicity to *Vibrio fischeri* for six alkanones, six aldehydes, and six alkenals, and a two-descriptor model for 19 compounds via multiple linear regression (MLR) analysis. After that, they carried out similar work for 15 haloalcohols, 11 halonitriles, 11 haloesters, 10 diones, and 63 compounds in the combined data set. These models yielded coefficients of determination *R*^2^ ≥ 0.846 [7].

Qin et al. introduced five QSTR models for *Vibrio fischeri* from 15 polar narcotics, 33 non-polar narcotics, 48 polar and non-polar narcotics, and 90 and 102 chemicals [8]. These models have the number of descriptors as 1–4 and the *R*^2^ as 0.88–0.79. Li et al. [9] and Wang et al. [10] built QSTR models for baseline compounds and less inert compounds to *Vibrio fischeri* with the single descriptor, logKow. The number of compounds in each model is 77 [9], 97 [10], 76 [10], and the *R*^2^ is 0.870 [11], 0.89 [10], and 0.79 [10].

The group of Sun and Wang utilized two molecular descriptors to correlate with the toxicity of 24 bromide-based ionic liquids (Br-ILs) against *Vibrio fischeri*. The model yielded a *R*^2^ of 0.954 [11]. Wang et al. proposed QSTR models of 17 alkylated aromatic hydrocarbons towards *Vibrio fischer* [12]. The model has 10 molecular descriptors and produced a *R*^2^ of 0.956. de Melo et al. predicted the toxicity of aromatic sulfones to *Vibrio fischeri* [13]. The QSTR models were based on two latent variables (or descriptors) and on the training sets of 35–41 aromatic sulfone chemicals, which yielded a *R*^2^ of 0.704–0.934 [13].

The afore-mentioned QSPR models belong to local models, focus on a particular chemical species, and have some limitations in predicting aquatic toxicity to *Vibrio fischeri*. Especially, some models have lower ratios between the samples and descriptors used in the QSTR models, reducing the statistical quality and leading to overfitting, that is, the QSTR models may have superior performance on the training data but perform poorly on the test set.

Recently, the group of Su established mechanistically based QSTR models for a larger data set of chemicals against *Vibrio fischeri* [5]. The number of chemicals and the *R*^2^ from the MLR models are, respectively, 172 and 0.787 for baseline chemicals, 133 and 0.737 for less inert chemicals, 57 and 0.801 for reactive chemicals, and 25 and 0.766 for acting chemicals. Although they are limited in application (i.e., the modes of action should be first determined for a chemical in relation to *Vibrio fischeri* before selecting these QSTR models), these QSTR models based on different modes of action have high prediction accuracy [5].

Support vector machine (SVM) has become an effective tool in nonlinear problems. The aim of this paper is to develop a global SVM classification model for the toxicity of a large data set including 601 chemicals towards *Vibrio fischeri*. Furthermore, it can be used for the prediction of the toxicity category of a chemical in relation to *Vibrio fischeri* without knowing its modes of action.

## 2. Results and Discussion

The binary logistic regression (BLR) analysis (Forward: Wald) in SPSS 19.0 was performed between 946 molecular descriptors and 601 acute toxicity data, log1/IBC_50_. Three BLR models were obtained, which, respectively, have the number of independent variables as one (MLOGP2), two (SM03_AEA (dm and MLOGP2), and three (SM03_AEA(dm), RDF145u and MLOGP2), and possess lower total prediction accuracies of 71.2%, 71.7%, and 71.5%. Stepwise MLR analysis in SPSS 19.0 was further carried out for these molecular descriptors and acute toxicity data in the total data set, resulting in a descriptor subset including ten molecular descriptors when the increasing coefficient of determination Δ*R*^2^ > 0.01 was set as the criterion for adding new variables. Table 1 and Table 2, respectively, show the characteristics and physical meaning of ten molecular descriptors selected in the MLR model.

As can be seen from Table 1, the *sig.* value of each descriptor is less than 0.001 (the default threshold being 0.05). Therefore, they are significant descriptors. Moreover, the variance inflation factor (*VIF*) of each descriptor is less than 5 (the default threshold being 10), indicating these descriptors independently reflect molecular structure information related to the toxicity, log1/IBC_50_.

BLR is essentially the same as MLR analysis in modeling relationships between dependent and independent variables [3]. The difference between them is that BLR deals with dichotomous dependent variables and MLR deals with continuous variables. Thus, the ten molecular descriptors selected in the MLR model were used as the descriptor subset to develop QSTR classification models for the toxicity (log1/IBC_50_) of chemicals to *Vibrio fischeri*. The results obtained from the BLR analysis are shown in Table 3. For the training set of the BLR classification model, the true positives (TP), false positives (FP), true negatives (TN), and false negative (FN) are, respectively, 176, 54, 187, and 34. The statistical parameters of specificity (=84.6%), sensitivity (=76.5%), and accuracy (=77.6% for Class − 1 and 83.8% for Class + 1) are above 76%. For the test set, the values of TP, FP, TN, and FN are 56, 22, 58, and 14, respectively. Its specificity (=80.6%), sensitivity (=71.8%), and accuracy (=72.5% for Class − 1 and 80.0% for Class + 1) are above 71%. The harmonic means of precision *F*_1score_ for the training set, test set, and total set are 80.0%, 75.7%, and 78.9%, respectively. Their Matthew’s correlation coefficients (MCC) between the experimental and calculated log1/IBC_50_ are 61.3%, 52.4%, and 59.1%, respectively. In addition, the total prediction accuracy is 80.5% for the training set (451 log1/IBC_50_), 76.0% for the test set (150 log1/IBC_50_), and 79.4% for the total data set (601 log1/IBC_50_).

The ten molecular descriptors selected in the MLR model were further used as input vectors to develop the SVM QSTR classification model from the 451 toxicity (log1/IBC_50_) in the training set by applying the LibSVM package on the MATLAB R2014a platform. By using a genetic algorithm, the SVM parameters *C* and *γ* were optimized under the conditions, with *C* being [100,500], *γ* being [0,1], *m*-fold cross-validation (*m* = 451), the maximum generation (maxgen = 200), the maximum population size (sizepop = 20), and the *ε*-insensitive loss function (*ε* = 0.01). In the end, the optimal SVM with the parameters, *C* = 253.8 and *γ* = 0.009, was obtained. For the training set in Table S1 in Supplemental Materials, the values of the parameters TP, FP, TN, and FN are 191, 30, 211, and 19, respectively. The optimal SVM model has an accuracy of 87.6% for Class − 1 and 91.0% for Class + 1, specificity of 91.7%, sensitivity of 86.4%, and the harmonic mean of precision *F*_1score_ of 88.6%. The leave-one-out (LOO) cross-validation accuracy is 72.9%.

Subsequently, 150 toxicities (log1/IBC_50_) in the test set were used to validate the optimal SVM model and the prediction results were shown in Table 3. The test set possesses the parameters TP = 57, FP = 17, TN = 63, and FN = 13, a prediction accuracy of 78.8% for Class − 1 and 81.4% for Class + 1, specificity of 82.9%, sensitivity of 77.0%, and a harmonic mean of precision *F*_1score_ of 79.2%. The total data set has TP = 248, FP = 47, TN = 274, and FN = 32, specificity = 89.5%, sensitivity = 84.1%, and *F*_1score_ = 86.3%. The MCC values of the training set, the test set, and the total data set are 78.3%, 60.1% and 73.8%, respectively, which are satisfactory, compared with the MCC results (61.3%, 52.4%, and 59.1%, respectively) from the BLR classification model. In addition, the prediction accuracy is 89.1% for the training set (451 log1/IBC_50_), 80.0% for the test set (150 log1/IBC_50_), and 86.9% for the total data set (601 log1/IBC_50_), which are higher than the results (80.5%, 76.0%, and 79.4%, respectively) from the BLR model. In addition to the accuracy, the parameters (specificity, sensitivity and *F*_1score_) of the SVM model are higher than that from the BLR model (see Table 3). Therefore, there are nonlinear relationships between the toxicities (log1/IBC_50_) and the ten molecular descriptors used, suggesting that it is reasonable to develop a SVM QSTR classification model for the toxicity (log1/IBC_50_) of chemicals to *Vibrio fischeri*.

Recently, the group of Lučić and Batista proposed a significant parameter (∆*Q*_2_) for validating the classification model quality [14,15]. The parameter ∆*Q*_2_ denotes the difference between the two-state accuracy (*Q*_2_) of the original model and the two-state random accuracy (*Q*_2,rnd_). For the BLR QSTR classification model, the training and test sets, respectively, possess *Q*_2_ values of 80.5% and 76.0%, *Q*_2,rnd_ values of 49.9% and 49.9%, and ∆*Q*_2_ values of 30.6% and 26.1%. The training and test sets in the SVM QSTR model, respectively, have *Q*_2_ values of 89.1% and 80.0%, *Q*_2,rnd_ values of 50.1% and 50.0%, and ∆*Q*_2_ values of 39.1% and 30.0%. Obviously, the ∆*Q*_2_ values from the SVM model are higher than that from the BLR classification model, meaning that the SVM model contributes a larger amount of useful information over the maximal level of random accuracy [16].

Many factors including the absorption, distribution, bioaccumulation, and metabolism of organic compounds in *Vibrio fischeri* influence the test results of toxicity, leading to the difficulty in investigating the mechanism of the toxicity of organic compounds towards *Vibrio fischeri*. The descriptor SpMax4_Bh(m) means the largest eigenvalue no. 4 of the Burden matrix was weighted by mass. It is calculated from a hydrogen-filled molecular graph with the Burden matrix Bh(w) and its largest positive eigenvalue. SpMax4_Bh(m) can reflect the molecular similarity/diversity in large databases. Table S1 shows that the compounds, cypermethrin (No. 117) and PR Toxin (No. 434), have high SpMax4_Bh(m) (3.914 and 3.346, respectively) and high log1/IBC_50_ (4.8 and 5.2, respectively) values. The reason is that they are typically reactive compounds: cypermethrin possesses benzylic activation with a good leaving group cyanide at an α-position, and PR Toxin has an epoxide.

AVS_B(p) belongs to 2D matrix-based descriptors and denotes the average vertex sum from the Burden matrix weighted by polarizability. Similar to SpMax4_Bh(m), the descriptor AVS_B(p) is derived from the Burden matrix Bh(w) of a hydrogen-filled molecular graph. As can be seen from Table S1, there are nine compounds, naphthalene (No. 112), indole (No. 412), 2-hydroxyfluorene (No. 142), 2-methylnaphthalene (No. 577), 1-methylnaphthalene (No. 582), xanthone (No. 100), biphenyl (No. 422), 1,3-dimethylnaphthalene (No. 586), with 1-naphthvlisocyanate (No. 428) having the highest AVS_B(p) values and possessing log1/IBC_50_ values ≥4.45 greater than the average value (log1/IBC_50_ = 4.02) of the total set. Due to the fact that the hydrogen atoms in benzene rings can be replaced by many different substituents, these compounds readily undergo nucleophilic aromatic substitution reaction, resulting in higher toxicity.

The descriptor MLOGP2 is derived from the squared Moriguchi octanol–water partition coefficient and widely used for QSTR models of compounds, especially for inert chemicals and less inert chemicals. A larger MLOGP2 expresses the compound with highly hydrophobic property, resulting in the accumulation of toxicant in *Vibrio fischeri* and causing toxicity [17,18]. Thus, the descriptor MLOGP2 is positively correlated with log1/IBC_50_ of chemicals to *Vibrio fischeri*.

The descriptor N-074 means the number of specific atom type, R#N or R=N-, with # here representing a triple bond and R being any group linked through carbon. In Table S1, there are 42 chemicals with N-074 > 0, their average log1/IBC_50_ value is 4.97, greater than the average log1/IBC_50_ value of 4.02 of the total set. These chemicals have reactive structural entities, for example, the chemicals of No. 600 (thiocyanates) and No. 601 (isothiocyanates), which can cause high log1/IBC_50_ values.

The descriptor B01[C-C] denotes the presence/absence of C-C at topological distance 1. In Table S1, there are only 12 chemicals without C-C at topological distance 1, that contain C-N, C-O, C-S, or only one C atom in the main chain, e.g., methanol (No. 1), formic acid (No. 464), dichloromethane (No. 6), chloroform (No. 313), tetromethylthiuramdisulfide (No. 149), bis(dimethylthiocarbamyl)sulfide (No. 278), and trichlorocyanuric acid (No. 293). The chemicals of Nos. 1, 13, 313, and 464 belong to non-polar narcosis and polar narcosis chemicals, while the chemicals of Nos. 149, 278, and 293 can form strong intermolecular force due to the strong electronegativity of heteroatoms, resulting in high toxicity.

The descriptor QXXm is the quadrupole X-component value/weighted by mass. It is associated with molecular polar and volume. For example, novobiocin (No. 393) has the highest value (QXXm = 336.668) and the highest molecular weight (=612.69). On the one hand, a larger QXXm indicates the corresponding chemical (or polar narcosis chemical) having strong polarity and leading to high toxicity compared with non-polar narcosis. On the other hand, a larger QXXm means the molecule possessing a larger volume, which hinders molecules penetrating the lipid bilayer of biological cell membrane and reduces the toxicity.

The descriptor CATS2D_04_NL denotes the number of pharmacophore point types of negative-lipophilic at a topological distance of four bonds. For CATS 2D descriptors, the atoms with a hydrogen bond donor (or acceptor), positively (or negatively) charged, or lipophilic pharmacophore points at topological distances not greater than nine bonds are taken into account. Table S1 shows that there are 41 chemicals with CATS2D_04_NL >0. Furthermore, all the chemicals contain acid groups. This descriptor is opposite to MLOGP2 mentioned above; a large CATS2D_04_NL indicates the chemical exhibiting strong hydrophilicity and resulting in lower log1/IBC_50_ value.

The descriptor C-016 is the number of =CHR groups in a molecule (R represents any groups linked through carbon). As can be seen from Table S1, 1,4-benzoquinone (No. 150) has the highest C-016 (=4). Its toxicity is very high (log1/IBC_50_ = 6.73), because this chemical enables a Michael-type addition of nucleophiles across the double bonds. In addition, some chemicals such as aldehydes and ketones (C-016 > 0) can have high toxicity when their α-position of the double bonds possesses a leaving group.

The descriptor DBI is the molecular branching index and reflects information on pairs of connected atoms in an H-depleted molecular graph. It is mainly related to molecular size and also sensitive to molecular branching. In Table S1, the chemical tetra-n-butyl-thiuramdisulfide (No. 510) has the highest DBI (=5.099) and lower toxicity log1/IBC_50_ (=3.83). The chemical has two terminal N atoms, each linking to two butyl groups. As stated above, it has a larger volume, hindering molecules across the lipid bilayer of the biological cell membrane, and reduces the toxicity. Other amines (e.g., tetrapropylthioperoxydicarbonicdiamide, No. 43) and amides (e.g., metolachlor, No. 486) have similar phenomenon.

The descriptor SM03_AEA(dm) is a spectral moment of order 3 from the augmented edge adjacency matrix weighted by the dipole moment. It encodes information about connectivity between graph edges and is related to spectral moments and fragment counts. Although a larger molecule in molecular weight or size usually has higher toxicity, too large a size will hinder molecular penetration and transport and decrease the toxicity. It is easy to understand that the chemical novobiocin (No. 393), with the highest value (SM03_AEA(dm) = 2.167) and the highest molecular weight (=612.69), only has a medium toxicity (log1/IBC_50_ = 4.38).

## 3. Materials and Methods

### 3.1. Experimental Data

There are 606 acute toxicity data, log1/IBC_50_ (the logarithmic form of 50% inhibition concentration of bioluminescence, in the unit of mol/L), of organic chemicals to *Vibrio fischeri* at the 15 or 30 min endpoint in [5]. After deleting the mixture (4-pyridylacetonitrile hydrochloride and four sodium compounds), 601 acute toxicity data were remaining and listed in Table S1 in Supplementary Materials. The toxicity data, log1/IBC_50_, are in the range of −0.25 to 7.12. The higher the log1/IBC_50_ value, the more toxic is the chemical to *Vibrio fischeri*. After sorting the log1/IBC_50_ values, it can be found that the samples with a log1/IBC_50_ value around 4.2 are sparse. Therefore, the threshold for Class + 1/− 1 was selected as 4.2, i.e., an organic compound is categorized as Class + 1 if its log1/IBC_50_ value is greater than 4.20, otherwise as Class − 1. Here, Class − 1 denotes the compound being nontoxic to *Vibrio fischeri* and Class + 1 means the compound being toxic towards *Vibrio fischeri*. The 601 organic compounds in the total data set were divided into a training set including 451 samples (Nos. 1–451 in Table S1 in Supplementary Materials) and a test set including 150 samples (Nos. 452–601 in Table S1) at the approximate ratio of 3:1. The training set includes 241 Class − 1 and 210 Class + 1, and the test set includes 80 Class − 1 and 70 Class + 1. From the training set of 451 log1/IBC_50_ data, a classification model was built, which was validated with the test set (150 log1/IBC_50_) that is different from the training set.

### 3.2. Molecular Descriptor Calculation

According to the chemical names in Table S1 in Supplementary Materials, each molecular structure was constructed with ChemDraw 19.0, and optimized with the molecular mechanics MM2 in Chem3D 19.0 at the default convergence level. After that, 4885 molecule descriptors were calculated for each molecule with the Dragon 6.0 [19], followed by removing those variables that equal a constant or possess partial correlation coefficients above 0.9. In the end, 946 descriptors were obtained for each molecule.

### 3.3. Support Vector Machine Algorithm

Rooted in the principle of structural risk minimization, SVM algorithms are widely used for both classification and regression [20,21]. They have excellent generalization capability on yet-to-be-seen data, even if a training sample size is small. In dealing with machine learning problems, its kernel functions are used to map the data points into a high dimensional feature space, then linear tasks can be implemented. In the SVM two-class problem, Equation (1) represents the separating hyperplane.

(1) $$f(x)=\sum_{i}^{n}\phi (x_{i})\omega +b$$

The slack variables *ξ_i_* and ξ_i_^*^ are introduced to guard against outliers. Then, the weight vector *w* and bias term *b* can be obtained by minimizing the objective function:

(2) $$\underset{w,b,\xi ,\xi *}{\min}J(w,\xi ,\xi^{*},b)=\frac{1}{2}{\left\|w\right\|}^{2}+C\sum_{i}(\xi_{i}+\xi_{i}^{*})$$

subject to:

(3) $$y_{i}-\varphi^{T}\left(x_{i}\right)w-b\leq \varepsilon +\xi_{i}$$

(4) $$\phi^{T}(x_{i})w+b-y_{i}\leq \varepsilon +\xi_{i}^{*}$$

The regularization constant (*C* > 0) is a tunable parameter. A larger C leads to more weight, decreasing the error. The *ε*-insensitive function is adopted in penalizing incorrect predictions:

(5) $${\left|f\left(x\right)-y\right|}_{\varepsilon }=\left\{\begin{array}{c} \left|f\left(x\right)-y\right|-\varepsilon \\ 0 \end{array}\right.,\;\begin{array}{c} \left(\left|f\left(x\right)-y\right|\geq \varepsilon \right)\\ \left(\left|f\left(x\right)-y\right|<\varepsilon \right) \end{array}$$

Then, Equation (1) becomes:

(6) $$f(x)=\sum_{i}^{n}(a_{i}-a_{i}^{*})\phi (x_{i})\cdot \phi (x)+b$$

The kernel function $K(x_{i},x)$ is introduced to deal with the nonlinear problem. The minimizing function can be written as:

(7) $$f(x)=\sum_{i}^{s}\left(a_{i}-a_{i}^{*}\right)K(x,y)+b$$

with $\alpha_{i},\alpha_{i}^{*}=0$, $\alpha_{i},\alpha_{i}^{*}\geq 0$. The coefficients $\alpha_{i},\alpha_{i}^{*}$ can be obtained by maximizing the quadratic programming problem:

(8) $$\mathrm{Max}:R(\alpha^{*},\alpha )=-\frac{1}{2}\sum_{i,j=1}^{n}\left(\alpha_{i}^{*}-\alpha_{i})(\alpha_{j}^{*}-\alpha_{j})K(x_{i},x_{j}\right)-\varepsilon \sum_{i=1}^{n}(\alpha_{i}^{*}+\alpha_{i})+\sum_{i=1}^{n}y_{i}(\alpha_{i}^{*}-\alpha_{i}).$$

(9) $$.S.T.\begin{array}{c} \sum_{i=1}^{n}(\alpha_{i}^{*}-\alpha_{i})=0\\ 0\leq \alpha_{i}^{*},\alpha_{i}\leq C \end{array}$$

In this work, the nonlinear problems above were solved with the Gaussian radial basis function:

(10) $$K(x_{i},x_{j})=\exp(-\gamma {\left\|x_{i}-x_{j}\right\|}^{2})$$

where *γ* is the kernel parameter. Similar to the regularization constant *C*, SVM models can be over-fitting or under-fitting on the training data when *γ* values are too large or too small [22,23]. The genetic algorithm (GA) and particle swarm optimization (PSO) algorithms are usually used for *C* and *γ* optimization. Due to the former being superior to the latter in the optimization speed, the genetic algorithm was used to find the optimal SVM parameters *C* and *γ* in this paper.

The parameters used to evaluate the performance of the classification models in this paper were defined as:

(11) $$\mathrm{sensitivity}=\frac{\mathrm{TP}}{\mathrm{TP}+\mathrm{FN}}$$

(12) $$\mathrm{specificity}=\frac{\mathrm{TN}}{\mathrm{TN}+\mathrm{FP}}$$

(13) $$F_{1\mathrm{score}}=\frac{2\mathrm{TP}}{2\mathrm{TP}+\mathrm{FN}+\mathrm{FP}}$$

(14) $$\mathrm{accuracy}\;\left(\mathrm{for}\;\mathrm{Class}+1\right)=\frac{\mathrm{TP}}{\mathrm{TP}+\mathrm{FP}}$$

(15) $$\mathrm{accuracy}\;\left(\mathrm{for}\;\mathrm{Class}-1\right)=\frac{\mathrm{TN}}{\mathrm{TN}+\mathrm{FN}}$$

(16) $$\mathrm{MCC}=\frac{\mathrm{TP}\;*\;\mathrm{TN}\;-\;\mathrm{FP}\;*\;\mathrm{FN}}{\sqrt{\left(\mathrm{TP}\;+\;\mathrm{FN}\right)\left(\mathrm{TP}\;+\;\mathrm{FP}\right)\left(\mathrm{TN}\;+\;\mathrm{FN}\right)\left(\mathrm{TN}\;+\;\mathrm{FP}\right)}}$$

(17) $$Q_{2}=\mathrm{accuracy}\;\left(\mathrm{for}\;\mathrm{Class}-1+\mathrm{Class}+1\right)=\frac{\;\mathrm{TP}\;+\;\mathrm{TN}}{\mathrm{TP}+\mathrm{FP}\;+\;\mathrm{TN}+\mathrm{FN}}\;\;$$

(18) $$Q_{2,\mathrm{rnd}}=\frac{\left(\mathrm{TP}\;+\;\mathrm{FN}\right)\left(\mathrm{TP}\;+\;\mathrm{FP}\right)+\left(\mathrm{TN}\;+\;\mathrm{FN}\right)\left(\mathrm{TN}\;+\;\mathrm{FP}\right)}{N^{2}}$$

(19) $$\Delta Q_{2}=Q_{2}-Q_{2,\mathrm{rnd}}$$

Here, TP being the true positives, FP being false positives, TN being true negatives, and FN being false negatives. The parameter ∆*Q*_2_ takes into account the level of two-state random accuracy and estimates the real model contribution to prediction accuracy above that level. A large ∆*Q*_2_ suggests that the classification model provides a significant level of useful information over the maximal level of two-state random accuracy [15,16].

## 4. Conclusions

Although many factors influence the toxicity log1/IBC_50_ of organic compounds to *Vibrio fischeri*, the ten molecular descriptors selected with MLR analysis were successfully used for the classification models by correlating with the structural information on the Michael-type addition of nucleophiles, molecular branching, molecular size, polarizability, hydrophobic, and so on. By the prediction of the optimal SVM model combined with the genetic algorithm, the training set (451 organics) has a prediction accuracy of 87.6% for Class − 1 (log1/IBC_50_ ≤ 4.2) and 91.0% for Class + 1 (log1/IBC_50_ > 4.2); the test set (150 organics) has the accuracy being 78.8% for Class − 1 and 81.4% for Class + 1, which are more accurate than those from the binary logistic regression model. The global SVM classification model is satisfactory, although it dealt with a larger data set (601) including the toxicity of organics to *Vibrio fischeri*.

## Figures and Tables

**Table 1:** Characteristics of ten molecular descriptors in MLR ^a^.

Descriptor	Coefficients	Std. Error	*t*-Test	*Sig.*	*VIF*
Constant	−2.029	0.510	−3.980	<0.001	/
SpMax4_Bh(m)	0.542	0.096	5.657	<0.001	4.167
AVS_B(p)	1.602	0.179	8.962	<0.001	3.264
MLOGP2	0.063	0.006	10.398	<0.001	1.342
N-074	0.448	0.095	4.708	<0.001	1.114
B01[C-C]	−1.463	0.0207	−7.063	<0.001	1.107
QXXm	−0.007	0.001	−6.043	<0.001	1.772
CATS2D_04_NL	−0.468	0.068	−6.902	<0.001	1.139
C-016	0.366	0.069	5.335	<0.001	1.097
DBI	0.292	0.060	4.891	<0.001	3.159
SM03_AEA(dm)	−0.179	0.039	−4.644	<0.001	1.428

^a^ Std. error being standard error; *Sig.* being significance test; VIF being variance inflation factor.

**Table 2:** Physical meaning and classes of ten molecular descriptors selected.

Physical Meaning	Class
SpMax4_Bh(m) means the largest eigenvalue no. 4 of Burden matrix weighted by mass and reflects molecular similarity/diversity on large databases.	Burden eigenvalues
AVS_B(p) is the average vertex sum from Burden matrix weighted by polarizability and associated with nucleophilic aromatic substitution reaction in benzene rings.	2D matrix-based descriptors
MLOGP2 denotes the squared Moriguchi octanol–water partition coefficient and describes molecular hydrophobic property.	Molecular properties
N-074 is the number of R#N/R=N-groups.	Atom-centered fragments
B01[C-C] reflects the presence/absence of C-C at topological distance 1.	2D Atom Pairs
QXXm means the quadrupole X-component value/weighted by mass and is related to molecular polar and volume.	Geometrical descriptors
CATS2D_04_NL denotes the CATS 2D negative-lipophilic at lag 04 and is associated with the type and the number of pharmacophore points.	CATS 2D
C-016 is the number of =CHR groups.	Atom-centered fragments
DBI is the Dragon branching index and correlated with molecular size and branching.	Topological indices
SM03_AEA(dm) is the spectral moment of order 3 from the augmented edge adjacency matrix weighted by dipole moment and reflects molecular fragment counts.	Edge adjacency indices

**Table 3:** Statistical results from the classification models ^a^.

Class	Exp.	Calc.		Acc.
		Class − 1	Class + 1	
Class − 1	241	187	54	77.6%
Class + 1	210	34	176	83.8%
(Overall Acc. = 80.5%) (Training set in BLR)		Spec.	Sen.	
		84.6%	76.5%	
		Class − 1	Class + 1	
Class − 1	80	58	22	72.5%
Class + 1	70	14	56	80.0%
(Overall Acc. = 76.0%) (Test set in BLR)		Spec.	Sen.	
		80.6%	71.8%	
		Class − 1	Class + 1	
Class − 1	321	245	76	76.3%
Class + 1	280	48	232	82.9%
(Overall Acc. = 79.4%) (Total set in BLR)		Spec.	Sen.	
		83.6%	75.3%	
		Class − 1	Class + 1	
Class − 1	241	211	30	87.6%
Class + 1	210	19	191	91.0%
(Overall Acc. = 89.1%) (Training set in SVM)		Spec.	Sen.	
		91.7%	86.4%	
		Class − 1	Class + 1	
Class − 1	80	63	17	78.8%
Class + 1	70	13	57	81.4%
(Overall Acc. = 80.0%) (Test set in SVM)		Spec.	Sen.	
		82.9%	77.0%	
		Class − 1	Class + 1	
Class − 1	321	274	47	85.4%
Class + 1	280	32	248	88.6%
(Overall Acc. = 86.9%) (Total data in SVM)		Spec.	Sen.	
		89.5%	84.1%	

^a^ The acronym ‘Exp.’ is for variable containing ‘experimental’ values, ‘Calc.’ for ‘Calculated’, ‘Acc.’ for ‘Accuracy’, ‘Spec.’ for ‘Specificity’, and ‘Sen.’ for ‘Sensitivity’.

## Data Availability

The data presented in this study are available in supplementary material.

## Supplementary Materials

The following supporting information can be downloaded at: https://www.mdpi.com/article/10.3390/molecules28062703/s1, Table S1: Molecular descriptors, experimental and predicted class labels.
